# Are Ecosystem Services Replaceable by Technology Yet? Bio-Inspired Technologies for Ecosystem Services: Challenges and Opportunities

**DOI:** 10.3390/biomimetics10110784

**Published:** 2025-11-19

**Authors:** Shoshanah Jacobs, Jindong Zhang, Emily Wolf, Elizabeth Porter, Shelby J. Bohn, Adam Maxwell Sparks, Marjan Eggermont, Mindi Summers, Claudia I. Rivera Cárdenas, Heather Clitheroe, Daniel Gillis, M. Alex Smith, Karina Benessaiah, Andria Jones, Adam Davies, Michael Helms, Dawn Bazely, Mark Lipton, David Dowhaniuk, Nyssa van Vierssen Trip, Nikoleta Zampaki, Peggy Karpouzou, Kristina Wanieck

**Affiliations:** 1 Department of Integrative Biology, College of Biological Science, University of Guelph, Guelph, ON N1G 2W1, Canada; eporte02@uoguelph.ca (E.P.); salex@uoguelph.ca (M.A.S.); 2 Biomimetics and Innovation Group, Faculty of Applied Informatics, Technology Campus Freyung, Deggendorf Institute of Technology, 94469 Deggendorf, Germany; jindong.zhang@th-deg.de (J.Z.); emilywolf113@gmail.com (E.W.); kristina.wanieck@th-deg.de (K.W.); 3 Department of Biology, Laboratory of Functional Morphology (FunMorph), University of Antwerp, 2000 Antwerp, Belgium; 4 Department of Biology, Faculty of Science, University of Calgary, Calgary, AB T2N 1N4, Canada; shelbyjbohn@gmail.com (S.J.B.); mindi.summers@ucalgary.ca (M.S.); 5 Department of Mechanical and Manufacturing Engineering, Schulich School of Engineering, University of Calgary, Calgary, AB T2N 1N4, Canada; meggermo@ucalgary.ca; 6 Instituto de Ciencias de la Atmósfera y Cambio Climático, Universidad Nacional Autónoma de México, Mexico City 04510, Mexico; claudia.rivera@ciencias.unam.mx; 7 Department of English, Faculty of Arts, University of Calgary, Calgary, AB T2N 1N4, Canada; hjclithe@ucalgary.ca; 8 School of Computer Science, College of Computational, Mathematical, and Physical Sciences, University of Guelph, Guelph, ON N1G 2W1, Canada; dgillis@uoguelph.ca; 9 Department of Geography, Environment and Geomatics, College of Social and Applied Human Sciences, University of Guelph, Guelph, Ontario, ON N1G 2W1, Canada; kbenessa@uoguelph.ca; 10 Department of Population Medicine, Ontario Veterinary College, University of Guelph, Guelph, ON N1G 2W1, Canada; aqjones@uoguelph.ca; 11 Interdisciplinary Studies, College of Arts, University of Guelph, Guelph, ON N1G 2W1, Canada; adam.davies@uoguelph.ca; 12 School of Mechanical Engineering, Georgia Institute of Technology, Atlanta, GA 30332, USA; michael.helms@me.gatech.edu; 13 Department of Biology, York University, Toronto, ON M3J1P3, Canada; dbazely@yorku.ca (D.B.); nyssa.trip@gmail.com (N.v.V.T.); 14 School of English and Theatre Studies, College of Arts, University of Guelph, Guelph, ON N1G 2W1, Canada; liptonm@uoguelph.ca (M.L.); ddowhani@uoguelph.ca (D.D.); 15 Faculty of Philology, School of Philosophy, National and Kapodistrian University of Athens, 15772 Athens, Greece; nikzamp@phil.uoa.gr (N.Z.); pkarpouzou@phil.uoa.gr (P.K.)

**Keywords:** manufactured ecosystems, biomimetics, bio-inspired design, anthropocene, climate adaptation, climate technology, more-than-human world, cultural ecosystem services, nature’s contributions to people, AI model

## Abstract

As ecological collapse accelerates under the pressures of anthropogenic climate change, adaptation strategies increasingly include technological proxies for nature’s functions. But can ecosystem services (ES) be meaningfully replaced by technology? Revisiting this urgent question first posed by Fitter (2013), we assess the extent to which bio-inspired design—particularly biomimetics—has advanced the capacity to support, enhance, or replace natural ES. We convened an interdisciplinary team to synthesize and refine a comprehensive list of 22 ecosystem services, integrating often-overlooked cultural and relational dimensions. Using this framework, we conducted a large-scale analysis of over 68,000 peer-reviewed publications from the biomimetics and bio-inspired design literature between 2004 and 2025, applying AI-assisted classification to evaluate whether, and how, these technologies map onto specific ES functions and benefits. Our findings reveal both promise and profound limitations. Bio-inspired research engages with 20 of the 22 ES, but over 78% of this work concentrates on five technologically tractable functions—biochemicals, disease regulation, waste treatment, fibre/hide/wood, and fuel. Foundational supporting and regulating services such as pollination, soil formation, and nutrient cycling are almost entirely absent. Moreover, only 3% of technologies described in the academic literature aim to support existing systems; the overwhelming emphasis on enhancement (39%) and replacement (58%) suggests a design paradigm skewed toward substitution rather than coexistence. Intangible, co-produced services—particularly those related to culture, identity, and meaning—remain outside the current reach of biomimetic design. This skew reveals a dangerous imbalance: while certain ES can be technologically approximated, the relational, emergent, and systemic qualities of ecosystems elude replication. Technological replacement must not become a substitute for preservation. Instead, bio-inspired design should be mobilized as a tool for adaptation that amplifies and protects the living systems on which human and more-than-human futures depend.

## 1. Introduction

The climate crisis is causing our natural ecosystem functions to break down. Pollinators are going extinct [1,2,3,4]; ocean oxygen is declining [5]; soil is eroding [6]; and human cultures that hold life-sustaining ecosystems knowledge are experiencing a severing of their relationships to the land (e.g., [7]). Efforts to mitigate the impacts of the ongoing Climate Crisis focus on reducing industrial activities to decrease global greenhouse gas emissions. The Paris Agreement stands as an ambitious landmark in the fight against climate change and in adapting to its effects. This legally binding international treaty on climate change, adopted by 195 Parties, aims to limit global warming to 1.5 degrees Celsius, compared to pre-industrial levels. However, as many scholars and critics agree, the Paris Agreement may not be sufficient, and the know-do gap between research and implementation widens as we fail to meet our global climate goals [8].

Though prevention and reduction efforts are ongoing, the pace of anthropocentric change [9] now requires strategies to cope and respond to rapid changes, forcing us to direct resources to adaptation and mitigation [10,11,12,13]. One adaptation strategy could be to deploy technological systems to support, enhance, restore, and if not possible, replace ecosystem services (ES)—that is, the benefits that people obtain from ecosystems, e.g., clean air, fertile soil, or potable water [14,15,16,17,18,19]. Adaptation strategies will become increasingly desirable, both economically and socially [20], as continued environmental destruction necessitates an immediate, worldwide, coordinated effort [8]. Efforts are underway across all regions and sectors at various scales; however, the resources supporting these efforts are not evenly distributed (e.g., [21]). For example, while conservation strategies attempt to reverse the loss of pollinators, researchers are simultaneously supplementing biological pollinators with technological ones (e.g., [22,23]). Individual technological solutions that replace specific ecosystem functions can be highly effective, particularly at local scales. These interventions are often necessary to address immediate ecological degradation. In light of the rapid scale and speed of changes experienced during the Anthropocene, our research must also consider broader, critical questions: what are the implications of designing entire ecosystems around the use of integrated technological proxies?

In failing or struggling ecosystems, bio-inspired design becomes an obligate tool to support, enhance, or replace ES [24,25]. This potential shift demands careful examination, especially as it becomes a more common response to large-scale environmental change. Bio-inspired design can mimic the diverse functions of living systems, and manufactured ecosystems will allow us to assess the ecological, social, cultural, economic, and political implications of this approach. We do this reluctantly, recognizing that the preservation of natural ecosystem functioning is paramount to technological enhancement or replacement. And recognizing that natural ecosystems are under tremendous strain, bio-inspired design becomes a necessary additional pillar to the precarious, ongoing balancing act of action, legislation, and restoration already underway.

A ‘manufactured ecosystem’ combines integrated visions of both nature and technology [25] through bio-inspired design [26,27,28,29,30,31,32,33] for supporting, enhancing, or replacing ES [19]. Manufactured ecosystems are products of intense innovation and artistic creativity, where technological proxies and practical solutions are developed in advance of an impending ecological collapse. Moreover, they can be created for various contexts and exist on a continuum with the natural world. For example, manufactured ecosystems can partially replace ecosystem services or fully replace all ES in the case of space exploitation and colonization, where a living biosphere is not present on moons or planets.

Our current capacity to envision manufactured ecosystems is insufficient because some of the required technologies do not yet exist. How do we synthesize oxygen for the entire human population without plants? How do we decompose organic material? Cycle water? If all our ES stopped today, we would be unable to replace the different ecosystem service categories with existing technological innovations, i.e., Cultural, Provisioning, Regulating, and Supporting functions [34].

Published 12 years ago, an article by Alastair Fitter asks, “Are Ecosystem Services Replaceable by Technology?” [16]. Fertilizers, sewage treatment plants, and insecticides may fit within the Ecosystem Technosphere; yet, Fitter (2013) rightly questioned the sustainability of these technologies, especially if they are required to operate in tandem during system-wide failures. When Fitter (2013) posed his question, RoboBees—autonomous flying microrobots capable of pollinating crops [35]—had not yet been invented. RoboBees technology meets the engineering requirements to achieve the biological function of pollination, but not the complex functionality of a living bee that provides other services, such as food production [36]. It is a biomimetic innovation [37,38] and serves as an artifact within a theoretically manufactured ecosystem [25]. Until we have adequately conserved or restored biodiversity, biomimetic innovation [26] might become necessary to enhance or fully replace lost ES. As we work to avert total climate-induced ecosystem collapse, there is potential for climate adaptation to be humanity’s greatest engineering feat.

Useful and correctly scaled technologies are predicated on the development of enhancement technologies [16]. We ask whether bio-inspired design facilitates the process of wide-scale climate adaptation by offering a systematic approach to transferring knowledge about ES to technological applications. Here, we ask again: if ES becomes replaceable by technology, what are the multifaceted implications of doing so?

## 2. Objectives

The present article takes steps toward answering that question by (1) identifying a comprehensive list of ES, and then (2) creating an extensive index of literature about technologies capable of performing, at least in part, the ecosystem service functions in question. This work takes inventory of our collective societal capacity for technologically supported climate adaptation. It systematically identifies gaps that require innovation, resources, and collaboration from designers, engineers, the social sciences and humanities, funding agencies, and governments, offering a foundational index to support scholarly and creative inquiry.

## 3. Methods

### 3.1. Ecosystem Services: Overview, Terminology and Understanding

The research team participated in structured discussions using four lists of previously published ES: Daily [39], Costanza [40], Fitter [16] (based on [41]), and the Millennium Ecosystems Assessment report [42]. These lists of ES have considerable overlap, but we noted important differences. Daily adopted an outcomes-centred model, emphasizing relatable human needs. For example, where Daily lists ‘mitigation of floods and droughts,’ Costanza lists ‘water regulation’ and ‘erosion control and sediment retention.’ Where Daily lists ‘biodiversity,’ Costanza lists ‘genetic resources.’ The Fitter list is based on Bateman et al.’s list of 20 ES, which is divided further into three categories to facilitate the development of climate adaptation strategies. ‘Supporting services’ are the least amenable to technology replacement. ‘Final services’ are those most amenable to technological replacement. ‘Goods’ are those for which there is already an existing economic market. The 2005 Millennium Ecosystems Assessment report provides a categorization framework useful for developing a climate adaptation strategy, emphasizing the cultural services ecosystems provide. Supporting, Provisioning, Regulating, and Cultural categories complement Fitter’s categories.

With representation from engineers, biologists, designers, literary scholars, writers, and communication and media specialists (Table 1), our research team held a series of three discussions on the four lists. For the first meeting, each core ES team member was asked to review the lists and arrive with their initial thoughts on comprehensiveness and relatability. During the meeting, the team discussed the similarities and differences between the lists and shared other resources (academic literature, government/policy documents, and works of fiction) they consulted and evaluated in developing their opinions. Our team engaged in debate and discussion to share and clarify individual interpretations. Through this, we learned the importance of intentionally using vocabulary and providing clear, specific definitions to ensure clarity in the meaning of different services for all individuals, regardless of their disciplinary background (e.g., some terms have different meanings in different disciplines; [43,44]. We also valued accessibility to a wide audience so that we could support multiple points of interest in the project.

Following the first meeting, our team assembled a preliminary list to share with the larger research team for comment and feedback. This highlighted the need to clarify the terminology and vocabulary used to describe and define ES further. We integrated various cultural services (i.e., the intangible benefits that people derive from nature, ranging from recreation to aesthetics, spirituality, and identity) [45,46], which have been underrepresented in discussions of ES, as they usually focus on ecological or economic functions. Previously, cultural ES have been presented as afterthoughts, appearing at the end of the list and rarely elaborated upon. For example, Daily includes ‘support for diverse human cultures’ and ‘aesthetic beauty and intellectual stimulation’ as the final two services, Costanza includes Recreation and Culture, Fitter includes ‘enjoyable environment’, and The Millennium Assessment identifies cultural services, including recreation, aesthetics, and spiritual value. The concept of nature’s contributions to people (NCPs) emphasizes the central role of culture in connecting people and nature. It highlights the diverse, multidimensional ways in which ecosystems support human wellbeing, identity, and livelihoods [36], while underscoring the role of humans—through culture and technology—in co-producing these services [47,48]. Many services, such as food in our present food and agri-food systems, cannot exist without human–nature interaction and technological mediation. In recognition of these interconnections emphasized by NCP, we included six cultural services in our list of 22 ES, significantly expanding upon previous conceptions of cultural services. This reflects nature’s co-production not only of ecological and economic benefits, but also of cultural and social meaning—for example, nature’s role in human identity formation and meaning-making [46,49].

### 3.2. Ecosystem Services: Literature Analysis

To explore whether existing technologies can replace ES, we screened the scientific literature for research situated at the intersection of ES and bio-inspired design. We focused on biomimetics, understood as the engineering of systems that imitate natural processes, because it is not merely a dominant strategy but an obligate design pathway for technologically reproducing the functions of living systems [25]. Unlike conventional engineering, which often abstracts or simplifies natural dynamics, biomimetics begins with the premise that evolutionary processes have already modified many ecosystem functions for resilience and adaptability. As such, biomimetic approaches are uniquely positioned to approximate the complexity, responsiveness, and integration found in nature. By identifying the extent and type of research in this field, we assess the technological landscape of ES replacements and reveal where such analogues are present, under development, or notably absent. The Web of Science Core Collection (WoSCC) was queried with the string “biomim* (Topic) OR bio*inspir* (Topic)” without any additional filters (20 May 2025). The search returned 69,063 records. After preliminary processing—cross-checking titles and DOIs (Digital Object Identifier) to remove duplicates—68,972 unique records were retained for analysis (see [50]).

To evaluate whether contemporary research in biomimetics (biom*)/bio-inspired design (bio*inspir*) has generated a theoretical or technological foundation capable of supporting, enhancing, or replacing natural ES, we conducted a text analysis of the biom* literature with a commercial large language model (LLM). The titles, keywords, and abstracts of these 68,972 publications were provided as input to the commercial LLM GPT-4.1 (gpt-4.1-14 April 2025) accessed via the OpenAI API). Using a prompt refined through multiple rounds of testing, the model was instructed to: (1) determine whether each publication explicitly describes a practical technological method that contributes to one or more of the 22 defined ES; and (2) if so, identify the relevant service(s) and categorize the contribution as “support”, “enhance”, or “replace”. The GPT-4.1 model was also asked to flag review articles—these were excluded from subsequent quantitative analyses. The LLM was not prompted to generate new content; only the provided prompts were used. For transparency and reproducibility, the full prompt used to steer GPT-4.1 is provided in Appendix A.

### 3.3. Challenges

Conducting the literature analysis proved challenging due to inconsistent terminology and disciplinary fragmentation. Terms like “biomimetics” and “bio-inspiration” are used differently across engineering, biology, and design fields, leading to a scattering of relevant research across disciplines and databases. Similarly, “ecosystem services” is a highly complex and inconsistently applied term, which created both false-positive and false-negative results in our search.

We began with structured keyword combinations such as “ecosystem service” AND “technology” AND “pollination,” expecting to retrieve well-known examples, such as the RoboBee project. Surprisingly, this and similar cases were not captured, suggesting that relevant research is often conducted and published under different labels or outside the ES discourse entirely. At the same time, many unrelated publications were retrieved, requiring manual review and classification to determine relevance.

The number of papers retrieved for each ecosystem service varied widely—from as few as 0 for Cultural Identity to over 11,000 for Biochemicals— which further complicated our analysis. Manual coding and blind reviewing became time-intensive and, in some cases, inconclusive, as the volume of material and lack of terminological coherence prevented a comprehensive or systematic overview. Despite significant investment in screening and coding, the resulting dataset did not reflect the field as anticipated, highlighting the need for a more unified vocabulary and indexing strategy in future research.

To overcome these challenges, we adopted a second approach: identifying specific technological keywords for each ecosystem service. However, this too proved problematic. There is no established or comprehensive mapping of technologies to ES, and any list we developed risked reflecting the biases and disciplinary assumptions of the researchers involved in this study.

The challenge intensified when we narrowed the focus to bio-inspired or biomimetic technologies. As with “ecosystem services,” the terms “bio-inspiration” and “biomimetics” are interpreted differently across fields. In some contexts, particularly in biotechnology, they are conflated with genetic engineering or bioutilization, rather than referring to the emulation of natural functions or forms. For instance, Fitter [16] references biotechnological interventions related to ecosystem function, but includes no examples of explicitly bio-inspired ES technologies. Conversely, none of the works cited in Fitter [16] appeared in our manual bio-inspiration searches, further illustrating the disciplinary disconnect. These challenges exemplify how knowledge can be constrained and squandered when it fails to transfer across disciplines.

**Figure 1:**
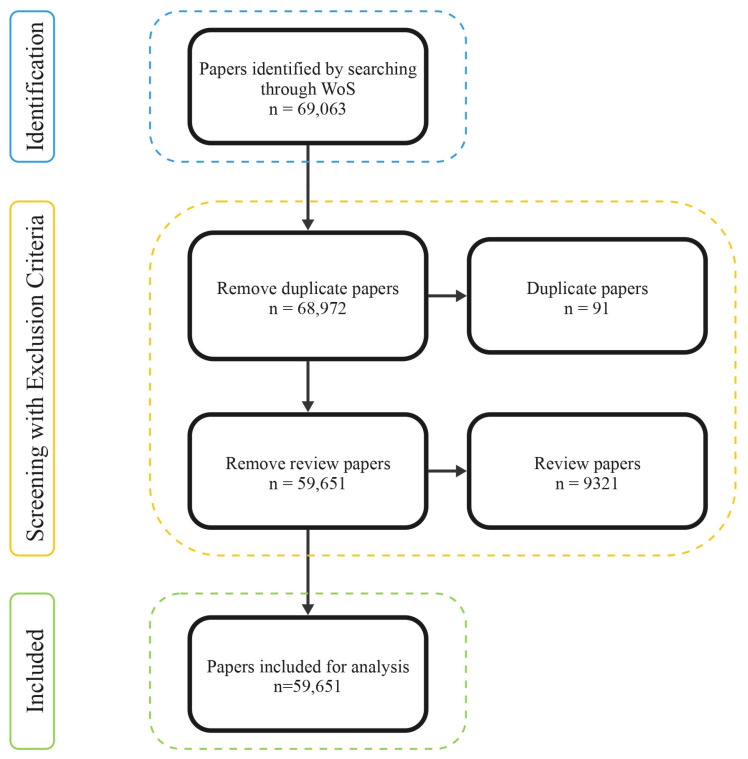
Overview of the literature search with exclusions. A total of 69,063 articles were initially identified; after the exclusion process, 59,651 were retained.

Given these limitations, we adopted a more computational approach. We began by compiling a large dataset of 68,972 articles (see [50]) using search terms beginning with “bio*inspir*” and “biomim*” to capture the language of biomimetics and bio-inspiration broadly. 9321 review articles were removed (Figure 1). We then used generative AI (GPT by OpenAI) to categorize these papers by the ES they address, employing a series of prompts developed with the ES framework described here. We acknowledge that this method has its limitations—AI will attempt to match publications to the ES category, even when no meaningful connection exists. Therefore, to assess reliability, we manually reviewed a subset of the first 100 AI-labelled articles. The mapping accuracy was 84.5%, suggesting the method holds promise for large-scale exploratory scans, despite requiring further validation and refinement.

## 4. Results

### 4.1. Ecosystem Services: Overview

**Figure 2:**
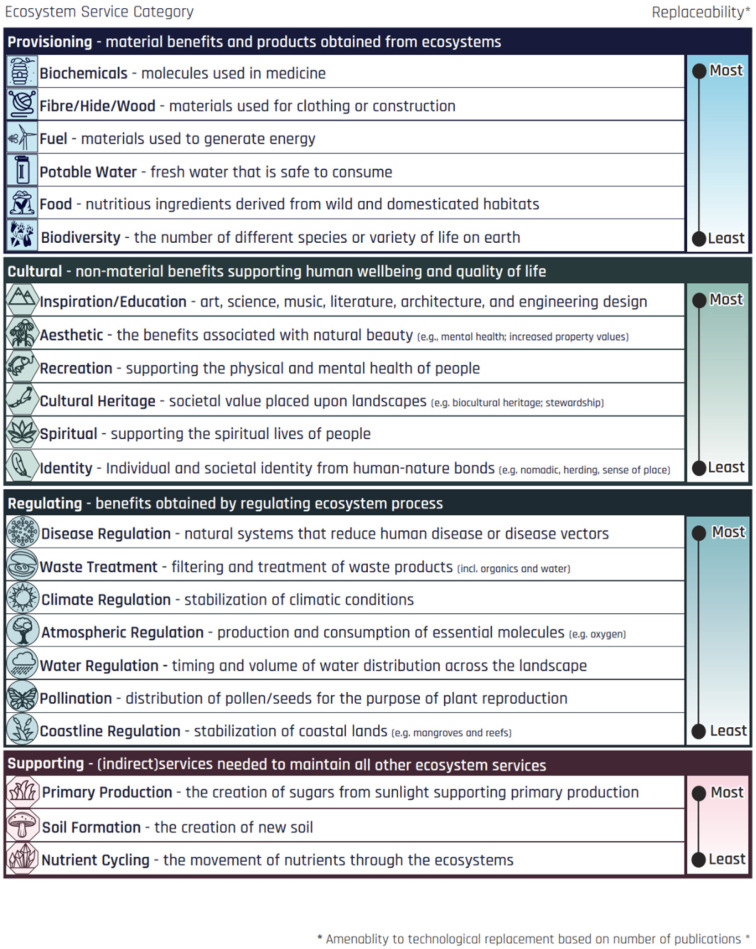
List of consolidated 22 Ecosystem Services. The list is modified based on Daily [39], Costanza et al. [40], Fitter [16], Bateman [41], and the Millennium Ecosystems Assessment [42]. Degree of replaceability was determined based on our research findings (see Figure 3).

Figure 2 presents the list of 22 consolidated ES, structured by the widely used four main categories from the Millennium Ecosystem Assessment: Provisioning, Cultural, Regulating, and Supporting. Provisioning services refer to the material benefits provided by ecosystems (e.g., food, raw materials), regulating include the natural processes such as climate regulation, water purification and pollination through which ecosystems maintain environmental conditions that support life, cultural (ecosystem) services relate to the non-material benefit that shape how people experience, value and connect with nature, while supporting services are the fundamental ecological processes—such as nutrient cycling and soil formation—that sustain all other services. Ecosystem services can sometimes be partially substituted by technology. Still, the degree of replaceability (or substitution) varies widely depending on the service and is often costly, localized, and less efficient than the services provided by nature. Provisioning services such as food and water can often be replaced or enhanced through agriculture, desalination, and biotechnology. Regulating services such as flood control or water purification can be mimicked by infrastructure and treatment systems, though typically at a higher cost and with lower efficiency. Cultural services—spiritual, recreational, and aesthetic benefits—are challenging to replace, as technology often cannot replicate the emotional and cultural connections to nature. Supporting services such as nutrient cycling and soil formation are virtually irreplaceable, as they underpin all other life-sustaining processes.

### 4.2. Ecosystem Service Technologies in Published Research Literature

#### Bio-Inspired ES Technologies Overview

**Figure 3:**
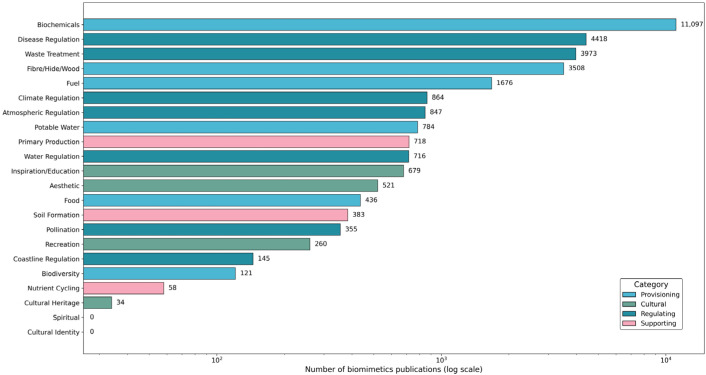
Literature coverage of 22 ecosystem-service classes within bio-inspired research. Horizontal bars show the number of research papers that explicitly address each service.

Of the 59,651 publications (Figure 1) analyzed, 53% (31,593 papers) were linked to at least one ES, and 20 of the 22 in our framework (Figure 2) were based on AI-assisted classification (Figure 3). This suggests that biomimetics and bio-inspired design research are already engaging with a substantial portion of the ES framework—either directly or indirectly. The only exceptions were Spiritual and Cultural Identity services, which were not directly associated in the dataset (Figure 3). This absence highlights a key limitation of current biomimetic research: while technical replication of ecological functions, such as pollination, air filtration, or nutrient cycling, is actively pursued, the cultural and symbolic dimensions of human–nature relationships remain largely unaddressed in technological discourse. This gap underscores the importance of broadening how we define and engage with “design,” particularly when attempting to replicate or replace ecosystems’ full range of contributions to people.

Within the ecosystem-service-linked biomimetics corpus, biochemical applications dominate, accounting for 35% of the 31,593 relevant articles, which focus on the extraction, synthesis, or structural emulation of bioactive compounds. This reflects the deep roots of biomimetics in medicinal chemistry, pharmacology, therapeutic design, and biomedical engineering. Disease regulation is the next most prevalent focus, comprising 14% of the dataset. These studies often explore biomimetic innovations such as antimicrobial surfaces, pathogen-resistant materials, and bio-inspired adhesives for medical use. Waste treatment follows closely at 13%, with a focus on enzyme-mediated plastic degradation, biofiltration technologies, and other environmentally responsive interventions. Materials inspired by the structure and function of natural fibres, hides, or wood account for 11%. In comparison, about 5% of the literature addresses energy-related applications, including bio-derived and bio-inspired fuel systems.

Together, these five services—biochemicals, disease regulation, waste treatment, fibre/hide/wood, and fuel—represented 78% of all biomimetics research linked to ES. This concentration suggests that the field has primarily targeted ES that are discrete, functional, and chemically or structurally replicable in laboratory or industrial settings.

In contrast, services arising from large-scale ecological processes remain marginal in the biomimetics literature. Climate regulation and atmospheric regulation each accounted for approximately 3% of the corpus, while water regulation comprised 2%. Foundational Supporting and Regulating services like pollination, soil formation, and nutrient cycling—critical to sustaining global food systems—collectively appear in just 2.5% of publications. This highlights a significant gap in the field’s current scope, underscoring the challenge of replicating complex, multi-scalar ecosystem functions with existing technological approaches.

### 4.3. Categorization: Support, Enhance, Replace

**Figure 4:**
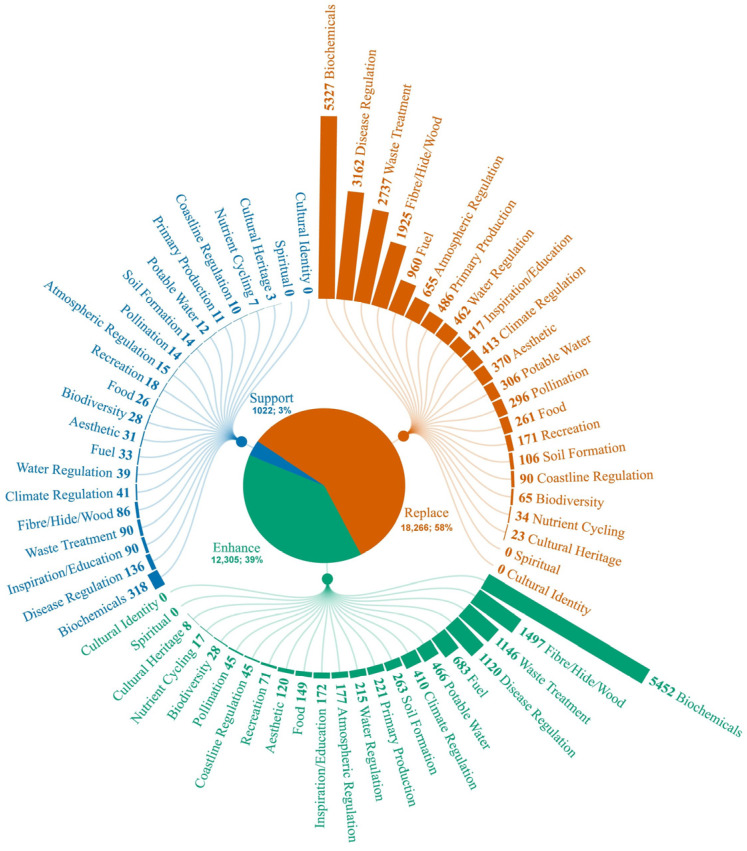
Distribution of bio-inspired research articles by technological trajectory and ecosystem service focus. The inner pie chart shows the proportion of the 31,593 ecosystem-service-linked publications that aim to replace (orange), enhance (green), or support the corresponding services (blue). Radial branches list the individual ES, and bar length represents the number of papers within that trajectory devoted to the service. Created with flourish.studio.

In the biomimetics corpus, 31,593 papers were assigned to one of three technological trajectories: support, enhance, or replace (Figure 4). Most publications fell into the replacement category (18,266 papers; 58%), indicating that more than half of the existing work aimed to deliver stand-alone technologies that could be viewed as substitutes for ecosystem functions. Enhancement papers are the second largest group (12,305 papers; 39%), while research whose stated aim is to support natural processes is comparatively underrepresented (1022 papers; 3%).

In the Replacement category, biochemicals (5327 papers; 29% of the category) lead well ahead of disease regulation (3162; 17%), waste treatment (2737; 15%), and fibre/hide/wood analogues (1925; 11%). A similar ranking is found within the enhancement category, where biochemical applications dominate (5452 papers; 44% of the category), followed by biomimetic fibre/hide/wood materials (1497; 12%), waste treatment (1146; 9%), and disease regulation (1120; 9%). Each remaining service contributes less than 5% to the enhancement total. Climate and atmospheric regulation together account for roughly 2% of publications in either replacement or enhancement categories, and water regulation remains below 3%. The Support category shows a flatter profile: biochemicals still provide the most significant single share (318 papers; 31% of the category), but several Regulating and Cultural services—such as inspiration/education (90; 9%), waste treatment (90; 9%), and fibre-based materials (86; 8%)—reach comparable proportions within this much smaller pool. In absolute terms, however, Support-oriented studies rarely exceeded 100 publications per service, and several functions—nutrient cycling, pollination, soil formation—are represented by fewer than 20 records each. Overall, large-scale regulating services (climate, atmosphere, water) and foundational agricultural functions (pollination, soil formation, nutrient cycling) together comprised less than 10% of the dataset.

## 5. Discussion

Our analysis revealed that existing bio-inspired research engages with 20 of the 22 defined ES. Spiritual and Cultural Identity were absent—likely a result of the way biomimetics and bio-inspired design [29] tend to translate observable form-and-function relationships into tangible artifacts. By definition, biomimetics aims to solve practical problems and holds the promise for sustainable solutions (ISO 18458). However, in the early stages of this scientific discipline [51], the focus was on solving technical problems associated with manufactured products, and this remains the primary objective [33]. Therefore, these fields prioritize services that can be instrumentalized, prototyped, and patented [16]; in short, technologies that can be marketed. Intangible, meaning-making services—those that foster worldview, belonging, or human transcendence—remain largely outside the scope of most biomimetic inquiry, even though the case for more holistic perspectives is already established (e.g., [52,53,54])

Where biomimetics is active, the focus is overwhelmingly on domains where molecular or component-level interventions are feasible [37]. The five most represented services—biochemicals, disease regulation, waste treatment, fibre/hide/wood, and fuel—make up 78% of the biomimetics literature linked to ES. By contrast, system-level services like pollination, soil formation, and nutrient cycling collectively account for just 2.5% of publications despite their foundational role in food security and ecosystem functioning. This disparity highlights an ongoing limitation in the field’s ability to address services that rely on emergent, interconnected ecological functions rather than discrete mechanisms.

When we disaggregate the literature by technological trajectory—support, enhance, replace—distinct patterns emerge. Only 3% of publications focus on supporting natural systems, while 39% aim to enhance, and 58% seek to replace, ecosystem functions. This imbalance reflects a dominant design logic that prioritizes substitution over coexistence. While technological innovation may offer opportunities for adaptation by substituting some ES, it cultivates a false sense of security—encouraging the belief that technological replacement absolves us of protecting the living systems upon which those services depend. This challenge is particularly evident in the abundance of technologies that focus on isolated functional analogues, which can often be commercialized, rather than considering the full ecological context. Replacement technologies may mimic a single output but rarely replicate the full complexity, interdependence, and resilience of the original system. RoboBees, for example, can pollinate crops but cannot replicate the broader ecological roles of living pollinators, such as providing a food source for insectivores, and are unlikely to appeal to spiritual and cultural needs in the same way that bees do. A shift toward supporting and enhancing living systems—not replacing them—should be viewed as essential, not optional, components of any adaptation strategy. This would better align bio-inspired design with complementary strategies such as ecosystem conservation and restoration, reinforcing the irreplaceable role of nature itself.

Although economic incentives often drive ES enhancement and replacement technology [16], we frame bio-inspired technologies as tools for climate change adaptation and mitigation. The goal is not to create fully manufactured ecosystems, but to prevent their necessity by offering solutions that protect and extend the functionality of natural systems. Moreover, because bio-inspiration promises environmentally sustainable solutions [55,56,57], the uptake of such technologies could align with broader efforts to safeguard biodiversity and habitat. Still, Fitter [16] cautions that for many ES—particularly those with broad spatial or temporal dynamics—no viable enhancement or replacement technologies exist, nor are they likely to emerge within the next 50 years. A core research question in this manuscript was whether bio-inspired design could provide a systematic method for transferring ecological knowledge into technological possibilities. Our analysis suggests that while bio-inspired design holds promise for targeted intervention, it currently lacks the scope, coherence, and systems-level orientation needed to address the full range of ES.

The most urgent threats facing humanity are not primarily geopolitical or economic, but ecological: for example, the accelerating collapse of services that provide healthy food, clean air, and safe water, and the presence of natural spaces that support spiritual and cultural practices. Our aim is not to promote a future in which nature is wholly engineered, but to provoke reflection on what would happen if we had to try. Realizing this vision will require international, interdisciplinary collaboration supported by agencies and governments willing to invest in novel, problem-solving approaches. While the field of bio-inspired design is still evolving [32,58], the increasing accessibility of biological knowledge—especially through AI-enabled search tools [59,60]—opens the door to a new era of design inspired by the logic of life itself.

What can be imagined for a manufactured ecosystem-found in the literary genre of science fiction-may eventually be realized, just as what can be imagined can prompt reflection on whether it should be manufactured. Econarratives of manufactured ecosystems shape the trajectory of humanity’s existence within the larger biosphere. The utility of a manufactured ecosystem lies in its ability to replace or support a natural ES effectively. Still, the endeavour must be situated within ethical, social, and economic realities [61]. Most ES are public goods; technologies, however, are often not. Replacing ES with proprietary or inaccessible systems risks shifting benefits away from the public and into private control. This underscores the need for globally coordinated, value-based research that prioritizes equity and collective wellbeing—ensuring that any future ecosystem, whether manufactured or not, serves the needs of all humanity.

Can we replace ES with technology yet? Our findings suggest: partially, and not without risk.

Technological replacement is not a complete answer; it is a contingency. The goal should not be to create an artificial ecosystem that substitutes for nature, but to develop tools that support adaptation while preserving the systems on which we still depend. Bio-inspired design, when developed responsibly, can offer powerful contributions to this future. However, if pursued without consideration for ecological complexity, public access, or social values, such technologies may not only alter how we receive ES but also who receives them.

Our conclusion is both cautionary and constructive: some ES can be mimicked; very few can be entirely replaced; none should be rendered optional.

## Figures and Tables

**Table 1:** Participants involved in developing the 22 Ecosystem Services list, their disciplinary expertise, and the mode of participation.

Participant	Discipline	Synchronous Participation	Asynchronous Participation
Claudia Rivera	Earth and Space Sciences		✔
Daniel Gillis	Mathematics and Statistics	✔	✔
Dave Dowhaniuk	Design and communication	✔	✔
Dawn Bazely	Plant ecology	✔	✔
Elizabeth Porter	Ecology	✔	✔
Emily Wolf	Biomimetics	✔	✔
Heather Clitheroe	Science Fiction		✔
Karina Benessaiah	Human-environment geography; Ecosystem Services; Sustainability Science	✔	✔
Kristina Wanieck	Biomimetics	✔	✔
M. Alex Smith	Biodiversity		✔
Marjan Eggermont	Engineering design		✔
Mark Lipton	Media studies	✔	✔
Michael Helms	Engineering		✔
Mindi Summers	Biology	✔	✔
Nikoleta Zampaki	Environmental Humanities		✔
Peggy Karpouzou	Literary Theory and Cultural Criticism		✔
Shoshanah Jacobs	Biomimetics and Education	✔	✔

## Data Availability

The raw data supporting the conclusions of this article will be made available by the authors on reasonable request.

## Appendix A. Prompt Used for GPT-4.1

You are an Ecosystem Service expert and a dedicated assistant designed to classify research articles (titles, keywords, abstracts given) based on the following instructions:

1. **Ecosystem Service Technology Analysis:**

Determine if the abstract describes a technological intervention that contributes to one or more of the following ecosystem services:

**Provisioning—Products obtained from ecosystems (Existing commercial market):**

- Biodiversity—The number of different species

- Food—Ingredients derived from wild and domesticated habitats

- Potable Water—Fresh water that is safe to consume

- Fuel—Materials used to generate energy

- Fibre/Hide/Wood—Materials used for clothing or construction

- Biochemicals—Molecules used in medicine

**Cultural—Benefits to quality of life and community (Existing commercial market):**

- Spiritual—Supporting the spiritual lives of people

- Recreation—Supporting the physical and mental health of people

- Aesthetic—The mental and physical health benefits of natural beauty

- Inspiration/Education—Art, music, literature, architecture, and engineering design

- Cultural Heritage—Value placed upon landscapes

- Cultural Identity—Societal identity regulated by the ecosystem (e.g., nomadic herding)

**Regulating—Benefits obtained by regulating ecosystem processes (Most amenable to technological replacement):**

- Atmospheric Regulation—Production and consumption of essential molecules (e.g., oxygen)

- Climate Regulation—Stabilization of climatic conditions

- Coastline Regulation—Stabilization of coastal lands (e.g., mangroves and reefs)

- Disease Regulation—Natural systems that reduce human disease or disease vectors

- Water Regulation—Timing and volume of water distribution across the landscape

- Waste Treatment—Filtering and treatment of waste products (incl. organics and water)

- Pollination—Distribution of pollen for the purpose of plant reproduction

**Supporting—Services that are not necessary for all other ecosystem services (Least amenable to technological replacement):**

- Soil Formation—The creation of new soil

- Nutrient Cycling—The movement of nutrients through the ecosystems

- Primary Production—The creation of sugars from sunlight

For this part:

**Decision:** Output “Y” if the abstract explicitly describes a practical technological method that contributes to one or more of these services; otherwise, output “N”.

**Category:**

If Decision is “Y”, choose **one** of the following:

- **”Support”** assists or maintains an existing natural process without intensifying it. Example: “Adding baffles so river flow still scours sediment but a little more efficiently.”

- **”Enhance”** significantly boosts the efficiency or scale of a natural process while still relying on that process. Example: “Embedding enzymes in a filter to double the nitrification rate; process still needs microbes.”

- **”Replace”** creates an artificial substitute that operates independently of the natural process. Example: “A photocatalytic panel that fixes nitrogen from air in total isolation from biological pathways.”

(If uncertain between Enhance and Replace, choose Enhance.)

Leave blank if Decision is “N”

**EcosystemService:** If Decision is “Y”, provide the exact ecosystem service from the list.

**Technology:** If Decision is “Y”, provide a concise short name for the technology used.

2. **Review Paper Detection:**

Determine if the abstract indicates that the article is a review paper. If the abstract contains phrases like “review”, “survey”, “meta-analysis”, or other similar indicators, then:

- **ReviewFlag:** Set to “review”.

Otherwise, leave this field blank.

**Output Format (Strict JSON Only)**

Your output must be in JSON format only, following this structure:

“Decision”: “Y” or “N”,

“Category”: “Enhance” or “Replace” (leave blank if Decision = “N”),

“EcosystemService”: “(exact ecosystem service from the list)” (leave blank if Decision = “N”),

“Technology”: “(concise short name of the technology)” (leave blank if Decision = “N”),

“ReviewFlag”: “review” or “”

}
